# Supplementary material for: Protocol for analysing the epidemiology of maternal mortality in Zimbabwe: A civil registration and vital statistics trend study
Source: PLoS One. 2021 Jun 3;16(6):e0252106. doi: 10.1371/journal.pone.0252106 (PMC8174727; doi:10.1371/journal.pone.0252106)
Supplement: Attachment — Submitted filename: Responses to reviewer comments.docx [file pone.0252106.s001.docx]

**To:**	The Editor

Plos One Journal

San Francisco, California, United States

**From:**	Reuben Musarandega

96 West Road, Avondale West, Harare, Zimbabwe

Tel: +263772773313;	[rmusara@g](mailto:rmusarandega@pedaids.org)mail.com

**Date:**	21 April 2021

**RE**: Submission of revised manuscript

We are pleased to resubmit our revised manuscript titled *“****Protocol for analysing the epidemiology of maternal mortality in Zimbabwe: a civil registration and vital statistics trend study.”****.*

In this resubmission letter, we have included our point-by-point response to the editorial and peer reviewer comments.

We sincerely thank the academic editors and peer reviewers for their comments which have improved the manuscript.

Yours faithfully,

**Reuben Musarandega**

*Lead and corresponding author*

**Response to Reviewers**

Journal Requirements:

**Comment 1:** Please ensure that your manuscript meets PLOS ONE’s style requirements, including those for file naming.

**Response:** We have updated the author information (lines 3-4 and 14-20), acknowledgements (lines 321-323), section headings of the paper (Abstract, Introduction, Methods and Materials and Discussion), citation of figures (Fig 1 & Fig 2) and naming of files according to journal requirements (Figures, Revised manuscript with tracked changes, revised manuscript clean version and responses to reviewer comments).

**Comment 2:** Please review your reference list to ensure that it is complete and correct.

**Response:** We updated some references with missing information. We did not identify any retracted references. If there is any, we will exclude it from our references.

Reviewer #1 Comments

**Comment 1:** Introduction: add the role of infection after es caesarean infection in Africa represent a high burden for maternal mortality and disability (see and cite maternal caesarean section infection (MACSI) in Sierra Leone: a case-control study. Epidemiol Infect. 2020 Feb 27;148:e40. doi: 10.1017/S0950268820000370)

**Response:** We have adopted this recommendation and added the relevant information in the discussion, paragraph 3, lines 284-287. To place this suggestion in full context, we added other causes identified in the literature.

**Comment 2:** Methods: very interesting

**Response:** We appreciated the comment and pleased that the study will contribute to maternal mortality research methodology.

**Comment 3:** Discussion: Discuss the role of the high dependency unit and the need in Africa of an area where critical patients can be cured (see and cite Epidemiology, Outcomes, and Risk Factors for Mortality in Critically Ill Women Admitted to an Obstetric High-Dependency Unit in Sierra Leone. Am J Trop Med Hyg. 2020 Nov;103(5):2142-2148 ) and how critical obstetric care are also economical for a health system with the help forex of NGO and other actors in health cooperation see and cite (Cost-Utility of Intermediate Obstetric Critical Care in a Resource-Limited Setting: A Value-Based Analysis. Ann Glob Health. 2020 Jul 20;86(1):82. doi: 10.5334/aogh.2907)

**Response:** We adopted this comment and accepted the first reference. However, we thought it fits better in the methods. Therefore, we added it in the section where we mentioned the source records for data from health institutions, in the methods section, lines 148-151.

**Comment 4:** Furthermore, discuss how, for example, during pandemic (ebola or other epidemics, see Impact of Ebola outbreak on reproductive health services in a rural district of Sierra Leone: a prospective observational study. BMJ Open. 2019 Sep 4;9(9):e029093. doi: 10.1136/bmjopen-2019-029093. ) women are always the most vulnerable group for all of these reasons; this paper is important.

**Response:** We adopted the comment and incorporated the suggested information in the discussion section, lines 288-290. Ebola has not affected Zimbabwe and Southern Africa at large. However, there has been a Cholera epidemic in Zimbabwe of similar magnitude and impact to Ebola. Hence we have added information on the Cholera epidemic in this section.

Reviewer #2 Comments

**Overall comment:** Overall, this protocol is well designed and complete.
Similarly, the programmed analysis is sound and well described.

**Response:** We appreciated the comment

Few minor revisions and questions:

**Comment 1:** We some interventions made to reduce maternal mortality in Zimbabwe between the two study period? It would be interesting to declare these interventions and perform a pre-post analysis

**Response:** We appreciated this comment and incorporated the suggestion. HIV interventions were already mentioned in the paper. However, we added funding for maternal and child health through two major multi-donor funds. We added this information in the discussion section, lines 299-301.

**Comment 2:** Please include Figure 1 and Figure 2 legend

**Response:** We have added the legends to the figures.

**Comment 3:** Could it be interesting to perform a competing risk analysis of death more than a “simple” logistic regression?

**Response:** We appreciated this suggestion and incorporated it in the data analysis section, lines 231-232. However, since competing risk analysis is a type of time-to-event or survival analysis, we are unsure if our study design and data will fit this type of analysis. We will explore it in study implementation.

**END OF COMMENTS**
